# On the efficiency of nature-inspired metaheuristics in expensive global optimization with limited budget

**DOI:** 10.1038/s41598-017-18940-4

**Published:** 2018-01-11

**Authors:** Ya. D. Sergeyev, D. E. Kvasov, M. S. Mukhametzhanov

**Affiliations:** 1 University of Calabria, DIMES, 87036 Rende, (CS) Italy; 2 Lobachevsky State University, Institute of Information Technology, Mathematics and Mechanics, 603950 Nizhni Novgorod, Russia

## Abstract

Global optimization problems where evaluation of the objective function is an expensive operation arise frequently in engineering, decision making, optimal control, etc. There exist two huge but almost completely disjoint communities (they have different journals, different conferences, different test functions, etc.) solving these problems: a broad community of practitioners using stochastic nature-inspired metaheuristics and people from academia studying deterministic mathematical programming methods. In order to bridge the gap between these communities we propose a visual technique for a systematic comparison of global optimization algorithms having different nature. Results of more than 800,000 runs on 800 randomly generated tests show that both stochastic nature-inspired metaheuristics and deterministic global optimization methods are competitive and surpass one another in dependence on the available budget of function evaluations.

## Introduction

Continuous global optimization problems arise frequently in many real-life applications^1–7^: in engineering, statistics, decision making, optimal control, machine learning, etc. A general global optimization problem requires to find a point x^*^ and the value *f*(x^*^) being the global (i.e., the deepest) minimum of a function *f*(*x*) over an *N*— dimensional domain *D*, where *f*(*x*) can be non-differentiable, multiextremal, hard to evaluate even in one point (evaluations of *f*(*x*) are expensive), and given as a “black box”. Therefore, traditional local optimization methods^8,9^ cannot be used in this situation. Among existing derivative-free global optimization methods two classes of algorithms can be marked out: stochastic metaheuristic algorithms (see, e.g.^4,6,10–15^) and deterministic mathematical programming methods^1–3,5–7,16–18^, etc. The former, due to their simplicity and attractive nature-inspired interpretations (genetic algorithms^6,10,12^, particle swarm optimization^15^, firefly algorithms^12,13^, etc.), are used by a broad community of engineers and practitioners to solve real-life problems whereas the latter are actively studied in academia due to their interesting theoretical properties including a guaranteed convergence. Historically, these two communities are almost completely disjoint: they have different journals, different conferences, and different test functions. Due to the hardness of global optimization problems and different nature of methods from these two groups, the problem of their comparison is very difficult and methods are collated on some dozens of test functions^1,2,15,16,19,20^ giving so a poor information and non reliable results. In order to bridge the gap between the two communities we propose a new efficient visual technique for a systematic comparison of global optimization algorithms having different nature. More than 800,000 runs on randomly generated 800 multidimensional test problems have been performed to compare five popular stochastic metaheuristics and three deterministic methods giving so a new level of understanding the tested algorithms. The test problems^21^ have been chosen because, after they have been randomly generated, the optimizer is provided with locations of the global minimum and of all local minimizers (this property has made the generator of these test problems very popular–it is used nowadays in more than 40 countries of the world). The knowledge of the global solution gives the possibility to check whether the tested method has found the global optimum. Since in practical problems the global solution is unknown and, therefore, it is not possible to check the quality of the obtained solution, it is very important to see how different methods are close to the global solution after their stopping rule has been satisfied.

In global minimization, problems where the *objective function f*(*x*) can have many local minima are considered and it is required to find the *global minimizer* (called also *global solution* or *global optimum*) x^*^ and the corresponding value *f* ^*^ such that

1 $${f}^{\ast }=f({x}^{\ast })\le f(x),\,\quad x\in D\subset {{\mathbb{R}}}^{N},$$

where *D* is a *search region*. In other words, among all the local minima (that are called *local solutions*) it is necessary to find the deepest minimum *f* ^*^ and its coordinates x^*^. It is well-known that a general continuous global optimization problem (1) is NP-hard^22–24^. This is true also, in particular, for problems (1) where the objective function *f*(*x*) satisfies the Lipschitz condition

2 $$|f(x^{\prime} )-f(x^{\prime\prime})|\le L||x^{\prime} -x^{\prime\prime} ||,\quad x^{\prime} ,x^{\prime\prime} \in D,\quad 0 < L < \infty ,$$

for a norm ||·|| with an unknown Lipschitz constant *L*. This condition means that any limited change in the parameters yields some limited changes in the values of the objective function. The assumption (2) can be justified by the fact that in technical systems the energy of change is always limited. In fact, this kind of problems can be very frequently met in practice (see^1–3,5,7,18^), in particular, in many engineering applications in which observations of the produced values of *f*(*x*) can be made, but analytical expressions of the functions are not available. For example, the values of the objective function *f*(*x*) can be obtained by running some computationally expensive numerical models, by performing a set of experiments, and so on. One may refer, for instance, to various decision-making problems in automatic control and robotics, structural optimization, engineering design, etc. The continuous global minimization problem (1) where *f*(*x*) satisfies (2) and can be non-differentiable, multiextremal, hard to evaluate even at one point, and given as a “black box” is studied in this paper.

In the traditional *local optimization*^9^, where strong assumptions on the structure of the objective function (such as convexity, continuity, differentiability, etc.) are made, these suppositions play a crucial role in the construction of any efficient search algorithm. In these cases, the dimensionality of the solved problem is often a measure of the goodness of optimization algorithms. In contrast, as was proved in^24^, if the only information about the objective function *f*(*x*) from the global optimization problem (1) and (2) is that it belongs to the class of Lipschitz functions and the Lipschitz constant *L* is unknown, there does not exist any deterministic or stochastic algorithm that, after a finite number of function evaluations, is able to provide an accurate *ε*-estimate of the global minimum *f* ^*^. That is why in this case instead of the theoretical statement (P1) *Construct an algorithm able to stop in a given time and to provide an ε-approximation of the global minimum f* ^*^ the more practical statement (P2) *Construct an algorithm able to stop after a fixed number M of evaluations of f*(*x*) *and to return the lowest found value of f*(*x*) is used. Under the latter statement, not the dimension of the problem (that is important in local optimization) but the number of allowed function evaluations (often called *budget*) becomes critical. In other words, when one has the possibility to evaluate *f*(*x*) *M* times (these evaluations are called *trials* hereinafter) in the global optimization problem of the dimension 5, 10 or 100, then the quality of the found solution after *M* evaluations is crucial and not the dimensionality of *f*(*x*). This happens because it is not possible to adequately explore the multi-dimensional search region *D* at this limited budget of expensive evaluations of *f*(*x*). For instance, if $D\subset {{\mathbb{R}}}^{20}$ is a hypercube, then it has 2^20^ vertices. This means that one million of trials is not sufficient not only to explore well the whole region *D* but even to evaluate *f*(*x*) at all vertices of *D*. Thus, the statement (P2) makes sense both because in practice the budget is always limited and because the problem under consideration is NP-hard.

As a result, the goal of global optimization methods is often to obtain a better estimate of *f* ^*^ and x^*^ given a fixed limited budget of evaluations of *f*(*x*). In fact, in global optimization the words “A method has solved a global optimization problem” very often do not mean that the global solution *f* ^*^ has been found. They mean just that the found solution was better than solutions found by other competitors (this is especially true for highly dimensional global optimization problems where the global solutions are unknown). That is why the possibility to compare the found solutions with the known global optimum offered by the generator of classes of test functions^21^ is very precious. It allows us not only to see that a solution *A* found by one method is better than a solution *B* found by another method, but to check whether these solutions are in a prefixed *ε*-neighborhood of the global optimum, i.e., to consider (P1) instead of (P2).

Let us describe now two groups of methods used in different communities and studied here. Metaheuristic algorithms widely used to solve (in sense of the statement (P2) discussed above) real-life global optimization problems have a number of attractive properties that have ensured their success among engineers and practitioners. First, they have limpid nature-inspired interpretations explaining how these algorithms simulate behavior of populations of individuals. Algorithms of this type studied here are: Particle Swarm Optimization (PSO) simulating fish schools^11^, Firefly Algorithm (FA) simulating the flashing behavior of the fireflies^13^, Artificial Bee Colony (ABC) representing a colony of bees in searching the food sources^14^, Differential Evolution (DE) and Genetic algorithms (GA) simulating the evolution on a phenotype and genotype level, respectively^4,6^. Other reasons that have led to a wide spread of metaheuristics are the following: it is not required to have a high level mathematical preparation to understand them; their implementation usually is simple and many codes are available for free; finally, they do not need a lot of memory working at each moment with only a limited population of points in the search domain. On the other hand, metaheuristics have some drawbacks including usually a high number of parameters to tune and absence of rigorously proved global convergence conditions ensuring that sequences of trial points generated by these methods always converge to the global solution x^*^. In fact, populations used by these methods can degenerate prematurely, returning only a locally optimal solution instead of the global one or even non locally optimal point if it has been obtained at one of the last evaluations of *f*(*x*) and the budget of *M* evaluations has not allowed to proceed with an improvement of the obtained solution.

Deterministic algorithms belonging to the second group of methods studied here are based on the knowledge that the objective function *f*(*x*) satisfies the Lipschitz condition (2). Lipschitz global optimization algorithms is a well-studied class of deterministic methods^1–3,5,7,18^. These methods are usually technically more sophisticated than metaheuristics, their implementation is not so easy, they require more memory and a higher mathematical preparation is necessary to understand and to use them. Commonly, they have a strong theory ensuring convergence to the global solution and a small number of control parameters allowing so their users to configure the search easily. Even though the Lipschitz constant *L* can be unknown, there exist several strategies for its estimation^2,3,5,7,18^ and one of the most frequently used techniques^16^ works with all possible values of *L* from zero to infinity simultaneously. All deterministic algorithms considered here use it. They are: DIRECT method^16^, its locally-biased version DIRECT-L^17^, and the algorithm^18^ based on adaptive diagonal curves (called ADC hereinafter).

How can one compare these two groups of methods? On the one hand, there exist several approaches for a visual comparison of deterministic algorithms (see, e.g., operational characteristics^25^, performance profiles^26^, data profiles^8^, etc.). However, they do not allow one to compare stochastic methods. On the other hand, comparison of metaheuristics often is performed on different collections of single benchmark problems^15,20,27^. As a result, the difficulty of test problems in collections can vary significantly leading sometimes to non homogeneous and, as a consequence, non reliable results. An additional difficulty consists of the fact that, due to a stochastic nature of metaheuristics, the obtained results cannot be repeated and have a character of some averages. Thus, the difficulties existing in performing a reliable comparison of these two groups of methods constitutes a serious gap between the respective communities. The goal of this paper is to start a dialog between them by proposing a methodology allowing one to compare numerically deterministic algorithms and stochastic metaheuristics using the problem statement (P1).

Instead of traditional comparisons executed just on several dozens of tests^1,2,15,16,19,20^ in this contribution more than 800,000 runs on 800 randomly generated test problems^21^ have been performed for a systematic comparison of the methods. In order to make this comparison more reliable, parameters of all tested algorithms were fixed following recommendations of their authors and then were used in all the experiments. One known and two novel methodologies for comparing global optimization algorithms are applied here: Operational Characteristics^25^ for comparing deterministic algorithms and new *Operational Zones* and *Aggregated Operational Zones* generalizing ideas of operational characteristics to collate multidimensional stochastic algorithms.

## Results

**Figure 1:**
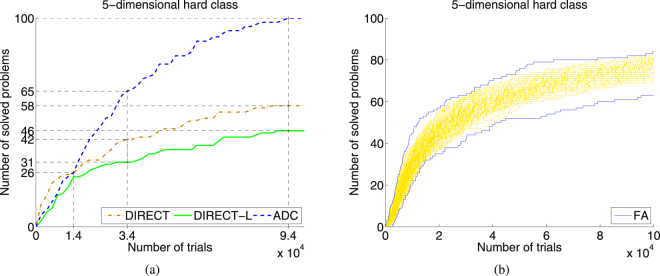
Construction of operational characteristics for deterministic methods and of the operational zone for metaheuristic Firefly Algorithm (FA) built on the hard 5-dimensional class of 100 GKLS test functions. (**a**) Operational characteristics for methods DIRECT, DIRECT-L, and ADC. After executing 34,000 trials DIRECT-L has solved 31 problem, DIRECT 42 problems, and ADC 65 problems; after performing 94,000 trials DIRECT-L has solved 46 problems, DIRECT 58 problems, and ADC all 100 problems. (**b**) The operational zone built using 10,000 runs performed by FA (100 runs for each of the 100 problems). The upper and the lower boundaries of the zone are shown as dark blue curves.

An operational characteristic^25^ constructed on a class of 100 randomly generated test functions is a graph showing the number of solved problems in dependence on the number of executed evaluations of the objective function *f*(*x*). To construct classes of test functions required to build operational characteristics, the popular GKLS generator^21^ of multidimensional, multiextremal test functions was used. This generator allows one to generate randomly classes of 100 test problems having the same dimension, number of local minima, and difficulty. The property making this generator especially attractive consists of the fact that for each function a complete information of coordinates and values of all local minima (including the global one) is provided. Here, 8 different classes from^18^ were used (see supplementary materials for their description and for definition of what does it mean that a problem has been solved). These classes and the respective search accuracies have been taken since they represent a well established tool used frequently to compare deterministic global optimization algorithms^18,28–31^. Fig. 1(a) shows operational characteristics for methods DIRECT, DIRECT-L, and ADC. Higher is a characteristic of a method with respect to characteristics of its competitors better is the behavior of this method. Operational characteristics allow us also to see the best performers in dependence on the available budget of evaluations of *f*(*x*). For instance, it can be seen from Fig. 1(a) that if the search budget is less than 14,000 possible trials than DIRECT method shows the best performance whereas for a budget superior to 14,000 the best method is ADC.

**Figure 2:**
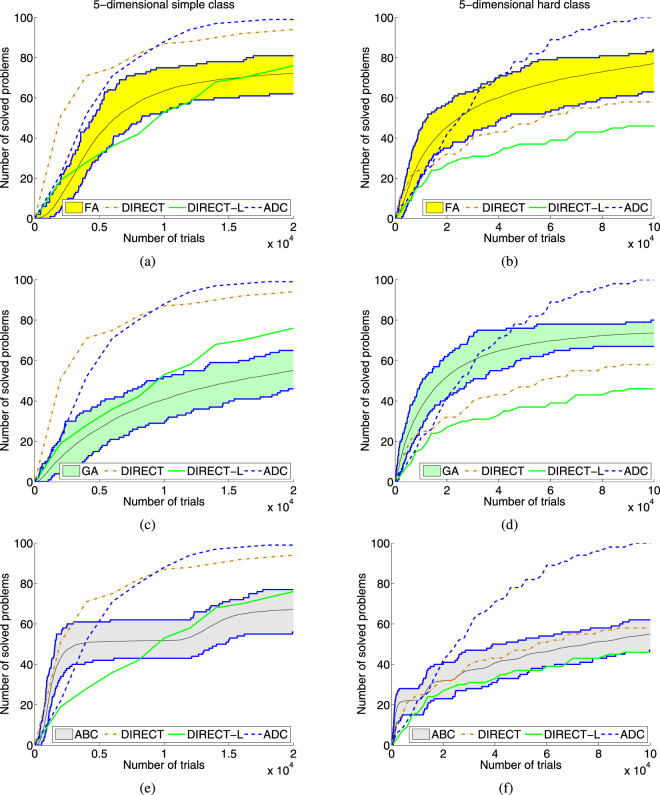
Operational characteristics built on two 5-dimensional classes of 100 GKLS test functions for deterministic methods DIRECT, DIRECT-L, and ADC and operational zones for stochastic metaheuristics Firefly Algorithm (FA), Genetic Algorithm (GA) and Artificial Bee Colony (ABC). (**a**) Operational characteristics for deterministic methods and the operational zone for FA for the simple 5-dimensional class. (**b**) The same as (**a**) for the hard class. (**c**) Operational characteristics for deterministic methods and the operational zone for GA for the simple 5-dimensional class. (**d**) The same as (**c**) for the hard class. (**e**) Operational characteristics for deterministic methods and the operational zone for ABC for the simple 5-dimensional class. (**f**) The same as (**e**) for the hard class.

Since operational characteristics cannot be used to compare stochastic methods, we propose in this paper a new methodology called *operational zones* that can be used for collating stochastic algorithms. To build a zone, a tested stochastic method should be launched *K* times (in our experiments each metaheuristic was launched *K* = 100 times for each of 100 test problems from each of 8 classes) with different randomly chosen populations (see supplementary materials for a detailed description of parameters of 5 tested metaheuristics) and a maximum number of trials *N*_*max*_ (in our experiments, *N*_*max*_ = 10^6^). Then, each run of a tested metaheuristic was considered as a particular method and its operational characteristic was constructed. The totality of all 100 operational characteristics form the respective operational zone (see Fig. 1(b) for an operational zone obtained by FA). Then, the upper and the lower boundaries of the zone (shown in Fig. 1(b) as dark blue curves) can be outlined (notice that they can contain parts of several characteristics) representing the best and the worst performances of the tested method, respectively. The graph for the average performance within the zone can be also depicted (see Fig. 2(b) where the average performance of FA is shown as a continuous black line inside the yellow operational zone).

Figure 2 shows results on the 5-dimensional simple and hard classes for metaheuristics FA, GA, and ABC (figures for these methods for other test classes as well as results for metaheuristics PSO and DE are given in the supplementary materials). Figure 2(a) and (b) compare, respectively, performance of the three deterministic methods and FA on the simple (with *N*_*max*_ = 2·10^4^) and the hard (with *N*_*max*_ = 10^5^) classes. The joint representation of operational zones together with characteristics offers a lot of visual information. It can be seen, for example, in Fig. 2(a) that operational characteristics of DIRECT and ADC are higher than the upper boundary of the zone of FA and, therefore, on this class deterministic methods have a better performance. Figure 2(b) shows that the lower boundary of the FA zone is higher than characteristics of DIRECT and DIRECT-L and, therefore, FA outperforms these competitors. If the budget is less than 30,000 trials (see Fig. 2(b)) than in average FA is better than ADC, as well. If the budget is higher than 40,000 trials than ADC behaves better since its characteristic is higher than the upper boundary of this FA zone. Notice also that Fig. 2(b) shows that after 10^5^ trials only the method ADC was able to solve all 100 test problems of the class. For the same two test classes, Fig. 2 presents operational zones for metaheuristics GA and ABC and for the three deterministic methods.

One can see also that in many runs metaheuristics got trapped into local minima and were not able to exit from their attraction regions producing so operational zones with long horizontal parts (see, e.g., Fig. 2(d) where metaheuristic GA works significantly better than DIRECT and DIRECT-L if the budget is less than 40,000 trials and then almost does not improve the number of solved problems remaining, however, always better than the two deterministic methods). This means that increasing the number of trials does not improve results in this case and it is necessary to restart metaheuristics. *Aggregated operational zones* proposed in this paper show what happens in this case. They are constructed as follows.

**Figure 3:**
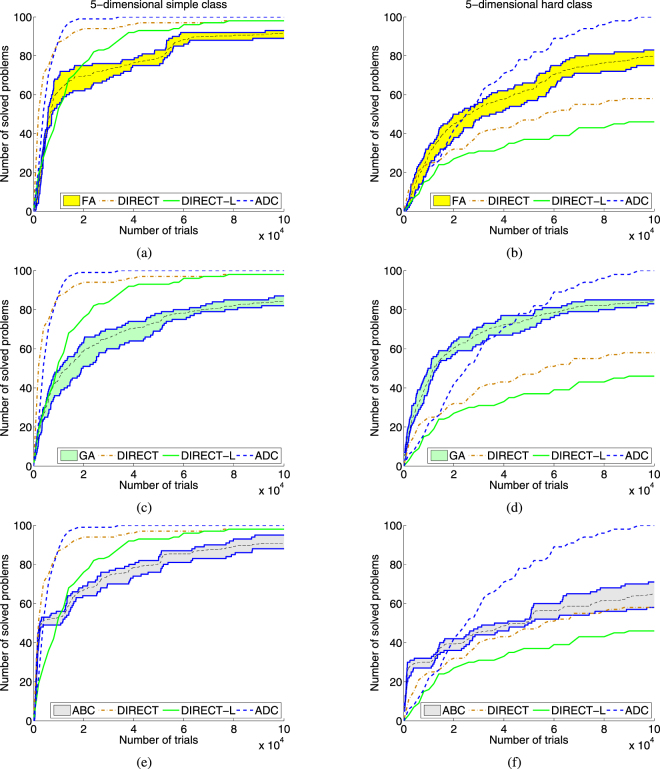
Aggregated operational zones for stochastic metaheuristics Firefly Algorithm (FA), Genetic Algorithm (GA), and Artificial Bee Colony (ABC) and operational characteristics for deterministic methods DIRECT, DIRECT-L, and ADC built on two 5-dimensional classes of 100 GKLS test functions. (**a**) Operational characteristics for deterministic methods and the aggregated operational zone for FA for the simple 5-dimensional class. (**b**) The same as (**a**) for the hard class. (**c**) Operational characteristics for deterministic methods and the aggregated operational zone for GA for the simple 5-dimensional class. (**d**) The same as (**c**) for the hard class. (**e**) Operational characteristics for deterministic methods and the aggregated operational zone for ABC for the simple 5-dimensional class. (**f**) The same as (**e**) for the hard class.

First, an algorithm is launched *K* times (*K* = 100 was used again here in order to have the same computational resources available for constructing operational zones) with an allowed number of trials *n*_*max*_ < *N*_*max*_ (in our experiments *n*_*max*_ = 50,000, *N*_*max*_ = 10^6^ for each metaheuristic). Then, for non-solved problems the algorithm is launched again with the same number *n*_*max*_ of allowed trials. Thus, if the algorithm did not solve a problem *p* in the first $n,1\le n < T,T=\lfloor {N}_{max}/{n}_{max}\rfloor ,$ runs but has solved it in the (*n* + 1)-th run in *t*, 1 ≤ *t* ≤ *n*_*max*_, trials then the number of trials to solve the problem *p* is equal to n*n_*max*_ + *t*. Otherwise, if the algorithm did not solve the problem *p* in *T* runs, then the number of executed trials for the problem *p* is set equal to the maximal allowed number *N*_*max*_ (in order to remind that more than *N*_*max*_ trials are required to solve this problem the mark “>10^6^” is used in Table 1). In this way, *T* runs are executed to complete the aggregated characteristic. Finally, *k* = *K*/*T* aggregated operational characteristics are used to build the aggregated operational zone in the same way as operational characteristics are used to construct an operational zone. The lower and upper boundaries are defined analogously. Fig. 3 shows results of experiments for the three deterministic methods and metaheuristics FA, GA, and ABC.

**Table 1:** Results of the experiments. For each test class the average number of trials required to solve all 100 problems is presented for each deterministic algorithm. For each metaheuristic method, the average number of trials required to solve each problem on 100 runs has been calculated, and the average of these 100 values is presented^†^.

N	Class	Metaheuristic algorithms (10,000 runs for each algorithm and class)			Deterministic algorithms (100 runs for each algorithm and class)		
		Genetic Algorithm	Artificial Bee Colony	Firefly Algorithm	DIRECT	DIRECT-L	Diagonal Algorithm
2	simple	>327452.5(2735)	2120.8	1190.3	198.9	292.8	176.3
2	hard	>370907.3(3149)	10366.2	>4299.6(3)	1063.8	1267.1	675.7
3	simple	>242231.7(1599)	10245.0	15269.2	1117.7	1785.7	735.8
3	hard	>412037.8(2874)	26254.2	>21986.3(1)	>42322.7(4)	4858.9	2006.8
4	simple	>150597.5(1290)	>57669.5(19)	23166.7	>47282.9(4)	18983.6	5014.1
4	hard	>247860.8(1900)	>150706.5(255)	40380.7	>95708.3(7)	68754.0	16473.0
5	simple	>237392.9(2208)	>38068.0(14)	>47203.1(16)	>16057.5(1)	16758.4	5129.9
5	hard	>249965.6(2311)	>230192.9(879)	>79555.2(38)	>217215.6(16)	>269064.4(4)	30471.8

^†^The record “>m(i)” means that the algorithm did not solve a global optimization problem *i* times in 100 runs ×100 problems (i.e., in 10,000 runs for metaheuristics and in 100 runs for deterministic algorithms). In this case, the maximal number of trials set to 10^6^ was used to calculate the average number of trials *m*.

It should be stressed that the aggregated operational zones allow one to emphasize better the potential of nature-inspired metaheuristics. In fact, the advantage of the aggregated zones with respect to the operational zones can be illustrated, for example, by situations shown in Figs 2(f) and 3(f). It can be seen from Fig. 2(f) that operational characteristics of deterministic methods DIRECT and DIRECT-L are located inside the zone of the metaheuristic ABC and, therefore, it is not possible to determine which of the three methods behaves better. In contrast, the aggregated zone of ABC is higher than the characteristics of both deterministic methods, i.e., it can be concluded that ABC outperforms them.

In order to see the advantages of the proposed methodologies for comparing methods, Table 1 constructed in a traditional way is shown. Due to the huge amount of data, only average results can be considered and included in Table 1. Notice that for deterministic methods and metaheuristics, due to the stochastic nature of the latter ones, different averages should be used: for metaheuristics the results on 10,000 runs for each class are used, whereas for the deterministic algorithms results on 100 runs (one run for each of 100 functions). This creates difficulties in comparing. For instance, which method is better on the 5-dimensional simple class: DIRECT or FA? On the one hand, DIRECT did not solve only one problem in 100 runs, demonstrating so success rate of 99%, whereas FA did not solve 16 problems in 10,000 runs, demonstrating, i.e., 99.84% of success. On the other hand, the average number of trials for DIRECT was only 16,057.5, while for FA 47,203.1 trials required in average. Moreover, Table 1 cannot give results for 50% or 75% of solved test problems, that can be also important. To see the detailed results, larger tables with hundreds of rows and columns should be used, complicating so the visual analysis of the results.

In contrast, operational zones very well present visually performance of tested methods giving the entire panorama of their behavior for different budgets. For instance, it can be seen from Figs 2 and 3 that metaheuristics perform very well on small budgets showing better results w.r.t. deterministic algorithms whereas the best algorithm for the higher budget on the used test classes is the algorithm ADC since it was able to solve all 100 test problems faster than other methods on both the classes. Even though this result can be also obtained from Table 1, the operational zones allow us to observe the performance of methods at all the stages of the search for each class. The average, the best, and the worst cases for each metaheuristic can be easily obtained from the graphs for any chosen number of trials. Moreover, the number of trials required to solve 50% (or 75%, 90%, etc.) of problems can be easily obtained and performance of methods is visualized clearly.

Let us see now another way for a statistical comparison of the two groups of algorithms using the same data. Let ${X}_{A}^{C}$ be a random variable describing the consumed percentage of the computational budget *N*_*max*_ performed by an algorithm *A* for solving a problem from the test class *C*. Let us consider the sample ${x}_{A}^{C}$ of 100 realizations of ${X}_{A}^{C}$ for *A* ∈ {*ADC*, *DIRECT*, *DIRECT* − *L*} and 100 × 100 = 10,000 realizations of ${X}_{A}^{C}$ for *A* ∈ {*FA*, *GA*, *ABC*, *PSO*, *DE*}, i.e., if, for instance, the algorithm ADC solved the 2-dimensional hard test problem number 5 after 574 trials, then ${x}_{ADC}^{2-hard}=\frac{574}{{10}^{6}}\times 100 \% =0.0574 \%$. Then, after the construction of the cumulative distribution functions ${F}_{{X}_{A}^{C}}(x)$, one can obtain the sampled distribution quantiles of ${X}_{A}^{C}$. For instance, in Tables 2 and 3, the sampled 25%, 50%, 75%, and 90% quantiles are presented for simple and hard classes, respectively. These results can be interpreted as follows. The 90%-quantile for the FA on the 5-dimensional simple class is 14.11%, whereas the same quantile for the ADC is 1.02%. This means that with the probability 90% FA will consume no more than 14.11% of the computational budget (i.e., no more than 141,100 trials), while ADC will consume no more than 1.02% of the computational budget (i.e., no more than 10,200 trials) to solve successfully the test problem. As it can be seen from Table 2, GA for the same test class and the same confidence level will consume 100% of the computational budget. This means that it cannot be claimed with the probability 90% that GA will solve the problem in the selected computational budget. However, it should be noted that with the probability 75% GA will resolve the test problem of the same class with no more than 9.24% of the computational budget (i.e., with no more than 92,400 trials).

**Table 2:** Results of the experiments. For each algorithm, quantiles *Q*_25_, *Q*_50_, *Q*_75_ and *Q*_90_ for the number of trials for simple test classes are presented.

*N*	*Q*	Metaheuristic algorithms (10,000 runs for each algorithm and class)					Deterministic algorithms (100 runs for each algorithm and class)		
		Differential Evolution	Particle Swarm Optimization	Genetic Algorithm	Artificial Bee Colony	Firefly Algorithm	DIRECT	DIRECT-L	ADC
2	25	0.07%	0.04%	0.13%	0.03%	0.05%	0.01%	0.01%	0.01%
	50	0.14%	0.08%	3.28%	0.05%	0.08%	0.01%	0.02%	0.02%
	75	0.26%	0.21%	99.53%	0.12%	0.14%	0.02%	0.04%	0.02%
	90	0.60%	95.74%	100.00%	0.69%	0.22%	0.04%	0.07%	0.02%
3	25	0.22%	0.29%	1.35%	0.09%	0.43%	0.02%	0.03%	0.05%
	50	0.44%	0.92%	4.83%	0.16%	0.84%	0.04%	0.06%	0.06%
	75	1.99%	3.60%	26.64%	1.06%	1.66%	0.15%	0.19%	0.10%
	90	35.70%	97.61%	99.83%	2.94%	2.91%	0.30%	0.57%	0.12%
4	25	0.17%	0.25%	0.24%	0.12%	0.32%	0.13%	0.23%	0.28%
	50	2.49%	1.04%	0.83%	1.34%	0.83%	0.50%	0.73%	0.41%
	75	89.48%	97.25%	3.78%	6.03%	2.69%	1.12%	3.07%	0.73%
	90	100.00%	100.00%	96.73%	15.89%	6.18%	2.21%	5.57%	0.89%
5	25	0.17%	0.27%	0.46%	0.12%	0.33%	0.06%	0.32%	0.21%
	50	0.29%	0.87%	1.56%	0.34%	0.62%	0.16%	0.93%	0.39%
	75	97.05%	4.04%	9.24%	3.73%	2.86%	0.64%	1.90%	0.64%
	90	100.00%	99.78%	100.00%	10.89%	14.11%	1.54%	3.54%	1.02%

**Table 3:** Results of the experiments. For each algorithm, quantiles *Q*_25_, *Q*_50_, *Q*_75_, and *Q*_90_ for the number of trials for hard test classes are presented.

*N*	*Q*	Metaheuristic algorithms (10,000 runs for each algorithm and class)					Deterministic algorithms (100 runs for each algorithm and class)		
		Differential Evolution	Particle Swarm Optimization	Genetic Algorithm	Artificial Bee Colony	Firefly Algorithm	DIRECT	DIRECT-L	ADC
2	25	0.09%	0.08%	0.43%	0.05%	0.07%	0.03%	0.06%	0.04%
	50	0.40%	0.26%	6.12%	0.23%	0.12%	0.11%	0.13%	0.06%
	75	2.96%	25.03%	99.05%	1.32%	0.22%	0.14%	0.18%	0.09%
	90	100.00%	100.00%	100.00%	2.87%	0.47%	0.22%	0.22%	0.12%
3	25	0.54%	0.61%	2.80%	0.13%	0.54%	0.04%	0.08%	0.12%
	50	14.88%	2.28%	18.64%	0.95%	1.11%	0.17%	0.20%	0.17%
	75	32.84%	99.91%	99.63%	3.20%	2.14%	0.37%	0.62%	0.27%
	90	100.00%	100.00%	100.00%	7.20%	4.02%	0.64%	1.25%	0.35%
4	25	93.83%	1.18%	0.48%	1.23%	0.63%	0.69%	0.67%	1.04%
	50	93.83%	12.42%	2.66%	6.16%	1.85%	1.61%	3.32%	1.51%
	75	100.00%	99.10%	26.78%	18.51%	4.93%	4.31%	11.31%	2.16%
	90	100.00%	100.00%	99.57%	41.96%	10.52%	13.40%	18.89%	2.90%
5	25	45.86%	0.59%	0.55%	1.27%	0.82%	0.89%	1.90%	1.31%
	50	95.77%	2.28%	1.82%	7.51%	2.46%	5.51%	18.29%	2.46%
	75	100.00%	21.34%	13.81%	30.74%	8.97%	15.09%	47.71%	4.44%
	90	100.00%	100.00%	100.00%	88.36%	21.57%	19.01%	69.96%	6.43%

On can see that the presented quantiles correspond to the results presented in Figs 1–3. In particular, the results presented in Tables 2 and 3 correspond to the average operational zones for each metaheuristic algorithm presented in Figs 2 and 3 (see also additional figures for the remaining test classes and the algorithms PSO and DE in the supplementary materials).

In conclusion, the proposed operational zones and aggregated operational zones allow one to compare effectively deterministic and stochastic global optimization algorithms having different nature and give a handy visual representation of this comparison for different computational budgets. Nature-inspired metaheuristics and deterministic Lipschitz algorithms have been compared on 800 of tests giving so a new understanding for both classes of methods and opening a dialog between the two communities. It can be seen that both classes of algorithms are competitive and surpass one another in dependence on the available budget of function evaluations.

## Electronic supplementary material

Supplementary Information
