# Supplementary material for: On the efficiency of nature-inspired metaheuristics in expensive global optimization with limited budget
Source: Sci Rep. 2018 Jan 11;8:453. doi: 10.1038/s41598-017-18940-4 (PMC5765181; doi:10.1038/s41598-017-18940-4)
Supplement: Supplementary file 1 — Supplementary Information [file 41598_2017_18940_MOESM1_ESM.pdf]

Supplementary notes:
On the efficiency of nature-inspired metaheuristics in expensive global optimization with limited budget

Ya.D. Sergeyev[1,2,*], D.E. Kvasov[1,2], and M.S. Mukhametzhanov[1,2]

[1]University of Calabria, DIMES, 87036 Rende (CS), Italy
[2]Lobachevsky State University, Institute of Information Technology, Mathematics and Mechanics, 603950 Nizhni Novgorod, Russia
[*]yaro@dimes.unical.it

Nature-inspired metaheuristic methods and their parameters

The following widely used in practice metaheuristic algorithms have been compared in our experiments with the well-known deterministic algorithms DIRECT, its locally-biased version DIRECT-L , and the algorithm based on adaptive diagonal curves (ADC).

- Genetic Algorithm (GA) is one of the basic nature-inspired evolutionary algorithms that simulates the evolution on a genotype level. This method uses in its work three main operators: crossover, mutation and selection. We used the real-coded genetic algorithm with the simulated binary crossover from http://www.egr.msu.edu/ kdeb/codes/rga/rga.tar. The parameters of the methods have been set as follows: the crossover probability was set to 0.95, mutation probabilities for binary and real coded variables were set to 0.2 and 0.25, respectively, in our experiments. $\lceil D/2 \rceil$ binary-coded variables and $\lfloor D/2 \rfloor$ real-coded variables were used. The chromosome length for binary-coded variables was set to 16. Finally, the parameter η of Simulated Binary Crossover (SBX) was set to 4.

- Artificial Bee Colony (ABC) simulates the intelligent foraging behavior of a honey-bee swarm. It provides a population-based search procedure in which candidates (foods positions) are modified by the artificial bees with time. The bee's aim is to discover places of food sources with higher nectar amounts (related to the objective function). In an ABC system, some of the artificial bees (employed and onlooker bees) choose food sources depending on the experience of themselves and their nest mates and adjust their positions. Other bees (scouts) fly and choose the food sources randomly without using experience. Therefore, during the work, the ABC system combines the local search (carried out by employed and onlooker bees) with the global one (managed by onlookers and scouts), thus attempting to balance exploration and exploitation processes. The number of employed bees was set equal to the number of onlooker bees and, thus, was equal to half population, i.e., one employed bee for each food source; the number of scout bees was set to 1. The basic ABC algorithm employs in its work only one control parameter *limit*. A food source is not anymore exploited and is abandoned by the bees when limit is exceeded for the source. The *limit* was set to $FoodNumber \cdot N$, where the *FoodNumber* is the number of food sources, i.e., the number of employed bees and N is the dimension of the problem. The realization of the algorithms was taken from http://sci2s.ugr.es/EAMHCO#Software.

- Firefly Algorithm (FA) belongs to the swarm intelligence algorithms as well and is inspired by the flashing behavior of fireflies. Each firefly (candidate solution) flashes its lights with some brightness (associated with the objective function). This light attracts other fireflies within its neighborhood. This attractiveness depends on the (Euclidean) distance r between the two fireflies and is determined by $\beta(r) = \beta_0 e^{-\gamma r^2}$, where β_0 is the attractiveness at $r = 0$. Hence, the search domain is explored by moving the fireflies towards more attractive neighbors (with some randomized moves allowed), thus improving the current best solution to problem. The attractiveness parameter β_0 was set equal to 1. The absorbtion coefficient γ was set equal to $1/\sqrt{l}$, where l is the average scaling factor of the problem (i.e., $l = \sum_{i=1}^{N}(b_i - a_i)/N$ for the search hyperinterval $\{x = (x_1, \ldots, x_N) | x_i \in [a_i, b_i]\}$. Finally, the randomization parameter α was set equal to 0.2. The realization of the algorithm was taken from https://github.com/firefly-cpp/Firefly-algorithm–FFA-.

Table 1. GKLS test classes. For each test class the radius of the convergence region ρ, distance between the paraboloid vertex and the global minimizer r, and the tolerance Δ from (1) are given.

N	Hardness	r	ρ	Δ
2	Simple	0.9	0.2	10^{-4}
2	Hard	0.9	0.1	10^{-4}
3	Simple	0.66	0.2	10^{-6}
3	Hard	0.9	0.2	10^{-6}
4	Simple	0.66	0.2	10^{-6}
4	Hard	0.9	0.2	10^{-6}
5	Simple	0.66	0.3	10^{-7}
5	Hard	0.66	0.2	10^{-7}

- Differential Evolution (DE) is another nature-inspired algorithm that solves a problem by maintaining a population of candidate solutions and creating new candidate solutions by combining existing ones with the usage of the crossover, mutation and selection operators, and then keeping the candidate solution with the currently best score (or fitness). The first control parameter F is a real constant which affects the differential variation (mutation) between two solutions and it was set to 0.7 in our experiments. The second control parameter is the value of crossover rate CR which controls the change of the population diversity and is between 0 and 1. It was set to 0.5 in the experiments. The mutation strategy "DE/Rand/2/exp" was used as one of the most powerful strategies. The realization of the algorithms was taken from http://www1.icsi.berkeley.edu/ storn/DenewC.zip.

- Particle Swarm Optimization algorithm (PSO) solves a problem starting from a population (swarm) of candidate solutions (particles) and moving these particles in the search domain according to the particle's position and velocity. At each iteration, a particle of the swarm updates its position by following the best local particle's position and the best solution of the whole swarm, thus guiding the swarm toward the best solutions. Cognitive ϕ_l and social ϕ_g parameters of PSO control the weighing on the personal (local) and swarm (global) experience, respectively; they were set to their default value 2.0 in our experiments. The inertia weight ω determines the influence of the previous velocity of a particle on its further velocity; we set it equal to 0.6 as for difficult and multimodal functions. The maximal velocity value was set equal to 15% of the longest side of the search domain. The algorithms was implemented in $C++$.

Additional results

In these supplementary materials, the operational zones for five tested metaheuristics are given for the lower dimensions $N = 2$, $N = 3$, and $N = 4$ and results for two algorithms that were not discussed in the paper (DE and PSO) are compared with the deterministic methods on 5-dimensional classes. Since it is reasonable to use aggregated operational zones only if the number of trials large enough can be performed, these zones are presented only on the 5-dimensional class for the algorithms presented in the paper (FA, ABC, and GA) which showed the best performance on the indicated classes of test problems.

In the experiments presented in the paper more than $800,000$ runs of the algorithms have been performed in total. In particular, each of 5 metaheuristics were launched 100 times on each of 800 test problems for both the approaches (operational zones and aggregated operational zones), giving so $800,000$ runs in total for the metaheuristic algorithms. Then, each of 3 deterministic algorithms were launched on each of 800 test problems, giving so $2,400$ runs in total. Thus, $802,400$ runs have been performed in total by all the algorithms.

GKLS test classes used in the experiments are presented in Table 1. Control parameters of the GKLS-generator required to produce classes of test problems are presented: the radius of the convergence region ρ, distance between the paraboloid vertex and the global minimizer r and the tolerance Δ (for details, see Sergeyev and Kvasov (2006)). Each problem was considered to be solved if an algorithm generated a point x' inside a hyperinterval with a vertex x^* and the volume smaller than the volume of the initial hyperinterval $D = [a, b]$ multiplied by an accuracy coefficient, i.e., if

$$|x'_i - x^*_i| \leq \sqrt[N]{\Delta}(b_i - a_i), \qquad 1 \leq i \leq N. \tag{1}$$

Finally, Table 2 presents results of the experiments for DE and PSO metaheuristics.

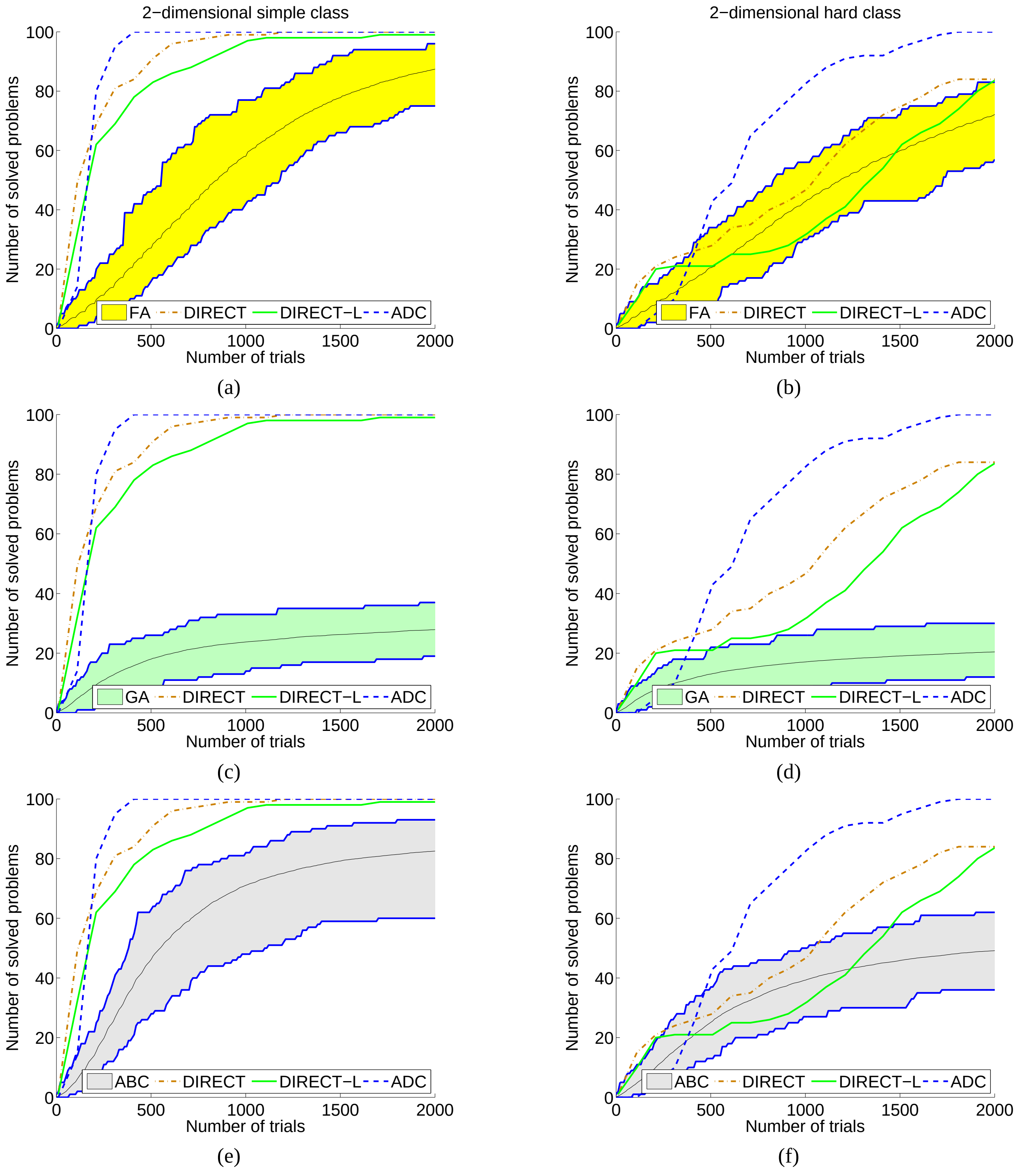

Figure 1. Operational characteristics and operational zones for the 2-dimensional GKLS classes for metaheuristics FA, GA, and ABC. **a,** Operational characteristics for deterministic methods and operational zones for FA are presented for the simple class. **b,** The same as (a) for the hard class. **c,** Operational characteristics for deterministic methods and operational zones for GA are presented for the simple class. **d,** The same as (c) for the hard class. **e,** Operational characteristics for deterministic methods and operational zones for ABC are presented for the simple class. **f,** The same as (e) for the hard class.

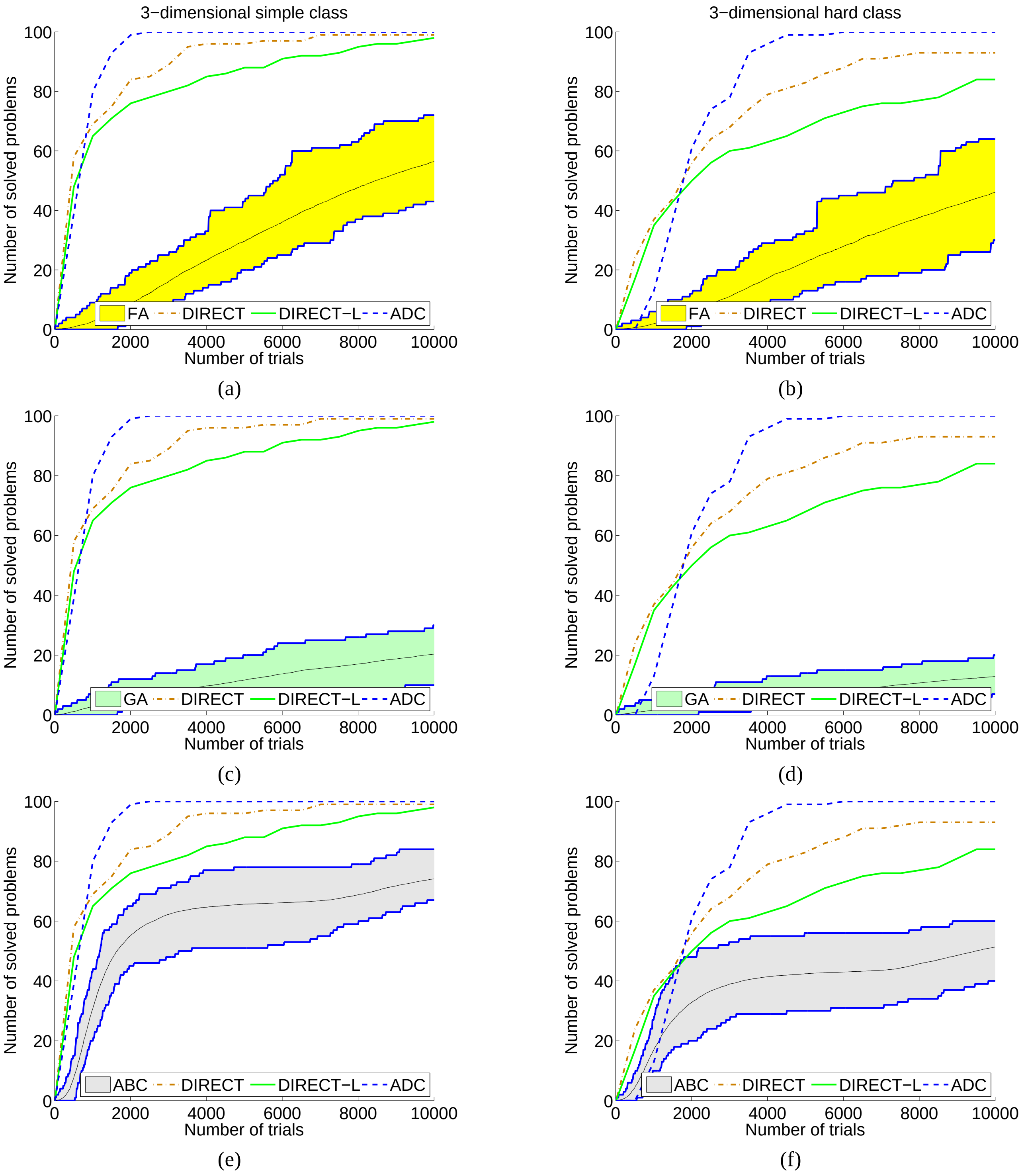

Figure 2. Operational characteristics and operational zones for the 3-dimensional GKLS classes for metaheuristics FA, GA, and ABC. **a,** Operational characteristics for deterministic methods and operational zones for FA are presented for the simple class. **b,** The same as (a) for the hard class. **c,** Operational characteristics for deterministic methods and operational zones for GA are presented for the simple class. **d,** The same as (c) for the hard class. **e,** Operational characteristics for deterministic methods and operational zones for ABC are presented for the simple class. **f,** The same as (e) for the hard class.

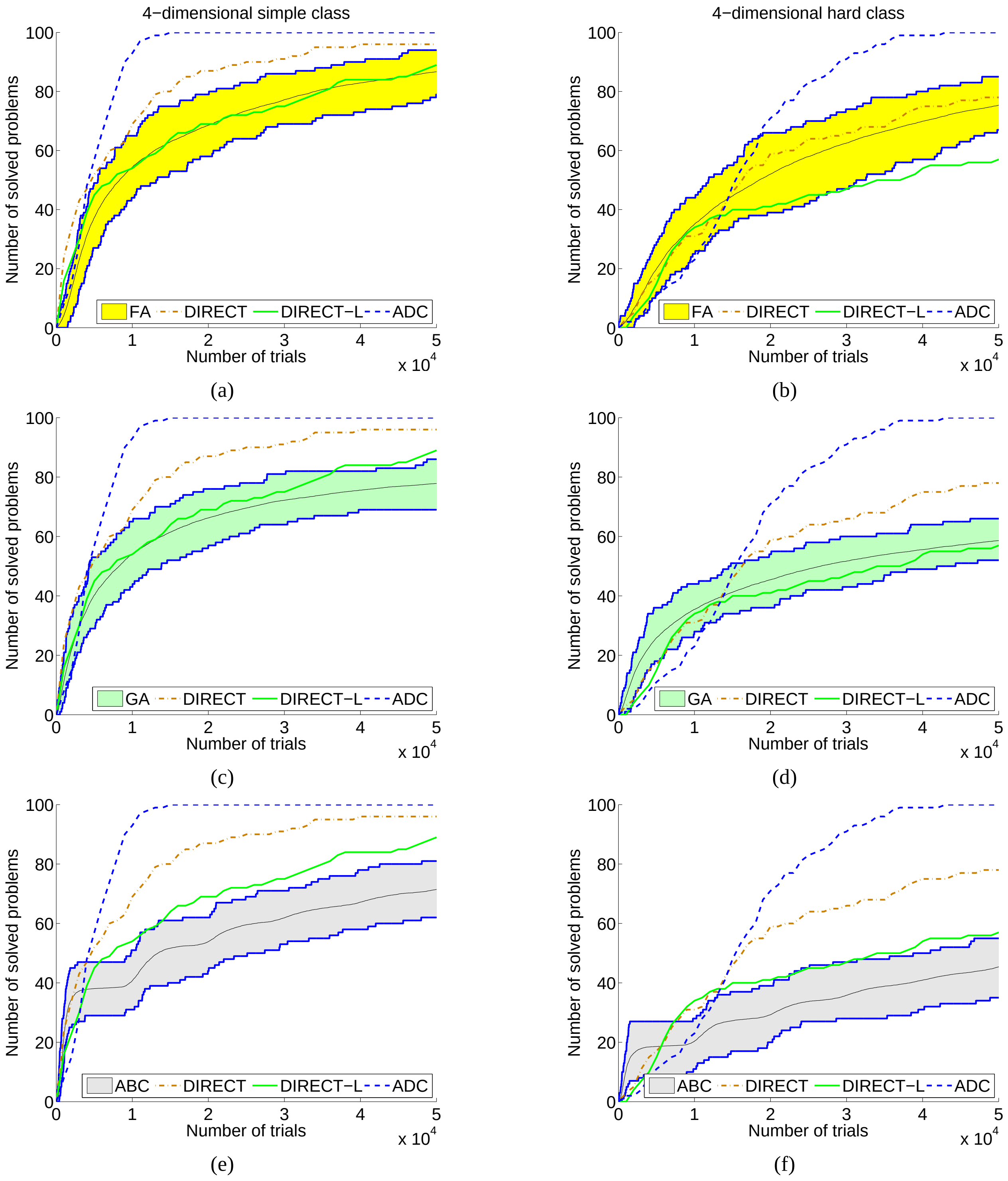

Figure 3. Operational characteristics and operational zones for the 4-dimensional GKLS classes for metaheuristics FA, GA, and ABC. **a,** Operational characteristics for deterministic methods and operational zones for FA are presented for the simple class. **b,** The same as (a) for the hard class. **c,** Operational characteristics for deterministic methods and operational zones for GA are presented for the simple class. **d,** The same as (c) for the hard class. **e,** Operational characteristics for deterministic methods and operational zones for ABC are presented for the simple class. **f,** The same as (e) for the hard class.

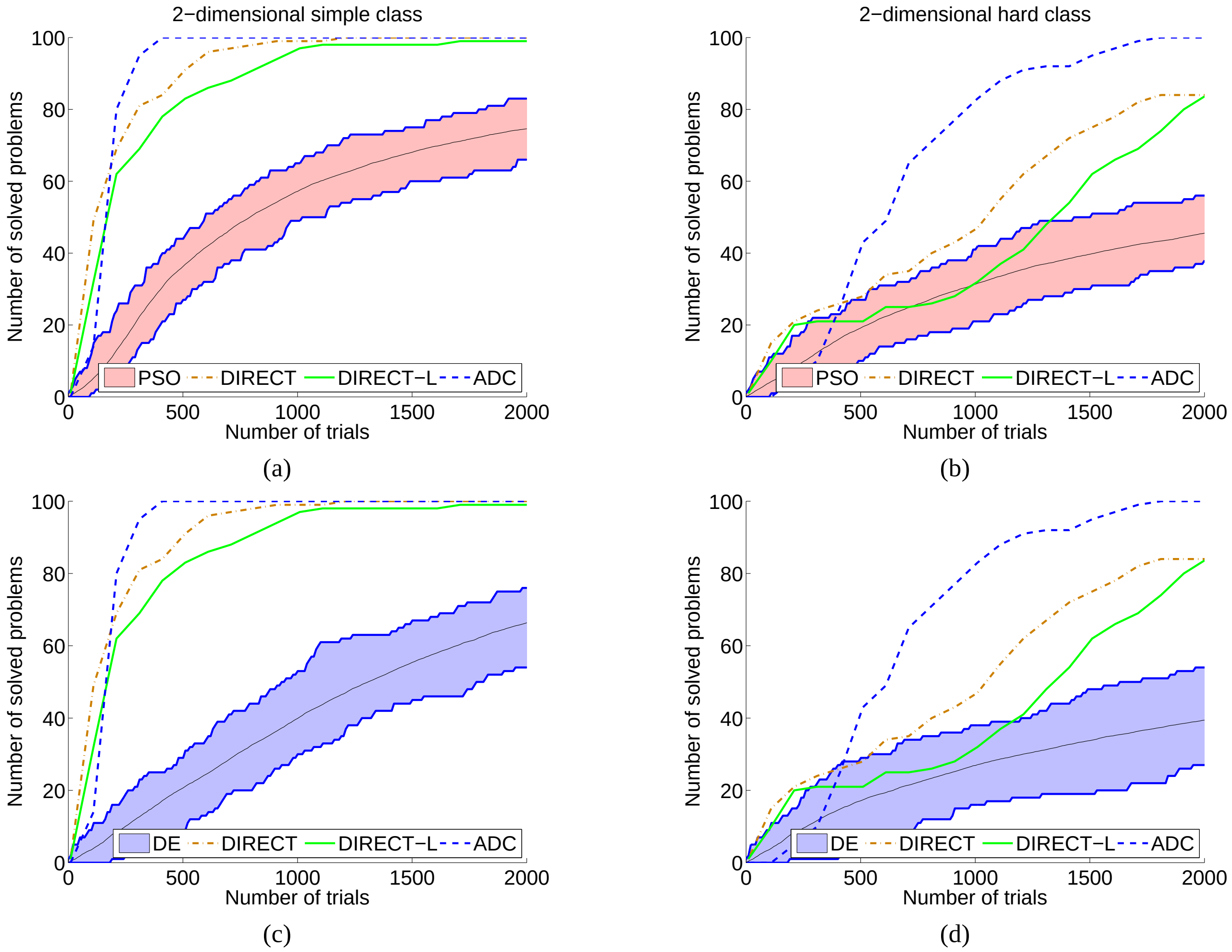

Figure 4. Operational characteristics and operational zones for the 2-dimensional GKLS classes for metaheuristics PSO and DE. **a,** Operational characteristics for deterministic methods and operational zones for PSO are presented for the simple class. **b,** The same as (a) for the hard class. **c,** Operational characteristics for deterministic methods and operational zones for DE are presented for the simple class. **d,** The same as (c) for the hard class.

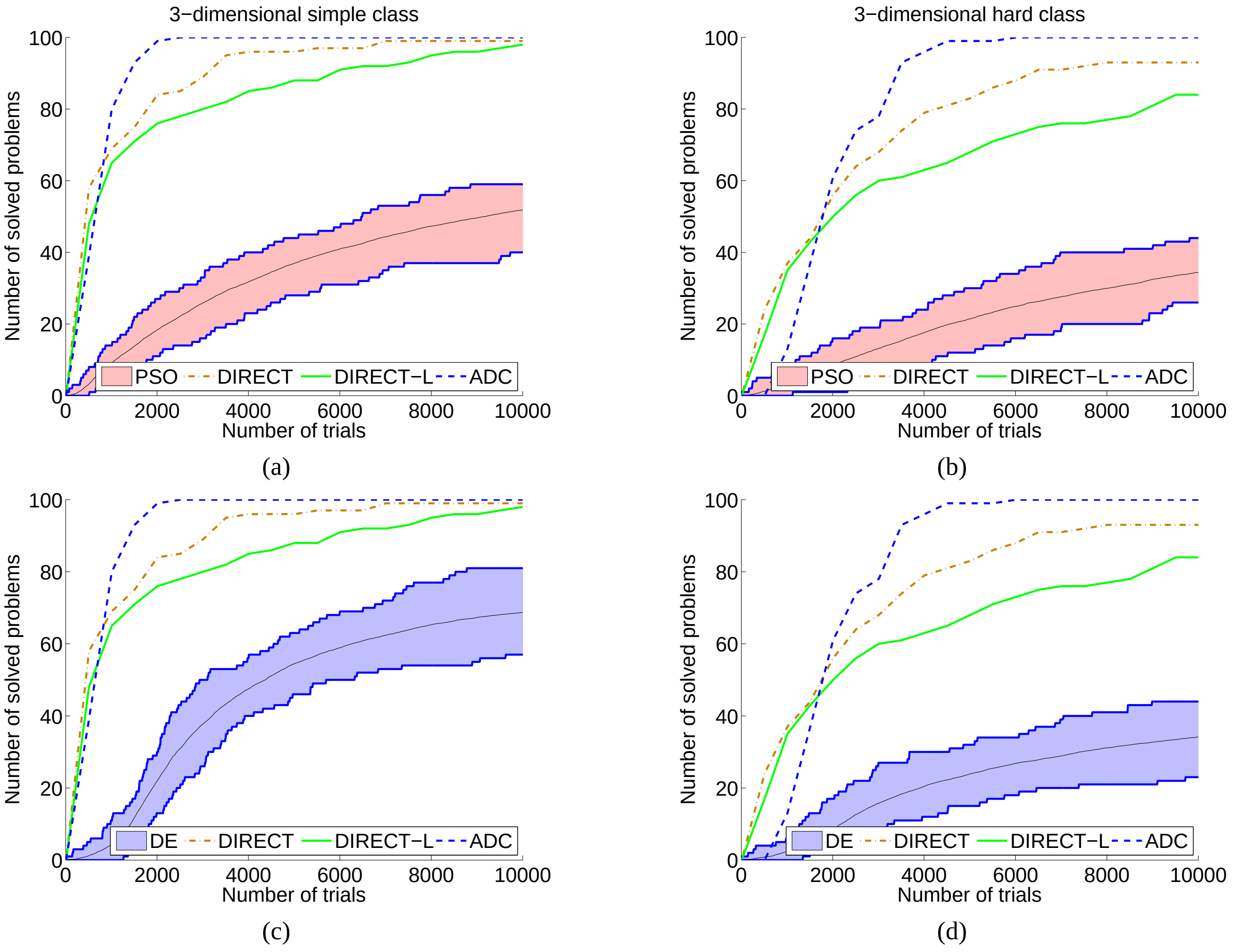

Figure 5. Operational characteristics and operational zones for the 3-dimensional GKLS classes for metaheuristics PSO and DE. **a,** Operational characteristics for deterministic methods and operational zones for PSO are presented for the simple class. **b,** The same as (a) for the hard class. **c,** Operational characteristics for deterministic methods and operational zones for DE are presented for the simple class. **d,** The same as (c) for the hard class.

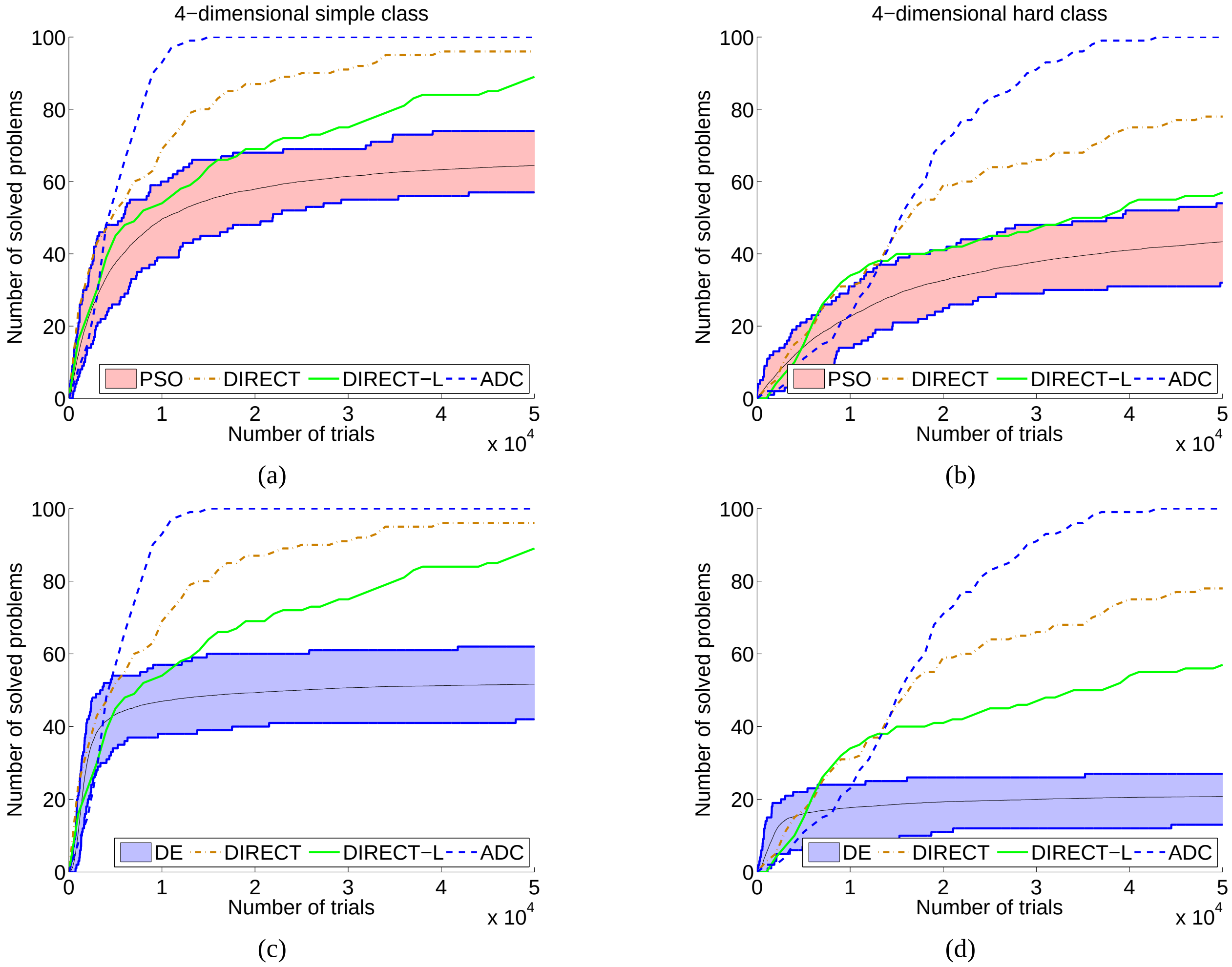

Figure 6. Operational characteristics and operational zones for the 4-dimensional GKLS classes for metaheuristics PSO and DE. **a,** Operational characteristics for deterministic methods and operational zones for PSO are presented for the simple class. **b,** The same as (a) for the hard class. **c,** Operational characteristics for deterministic methods and operational zones for DE are presented for the simple class. **d,** The same as (c) for the hard class.

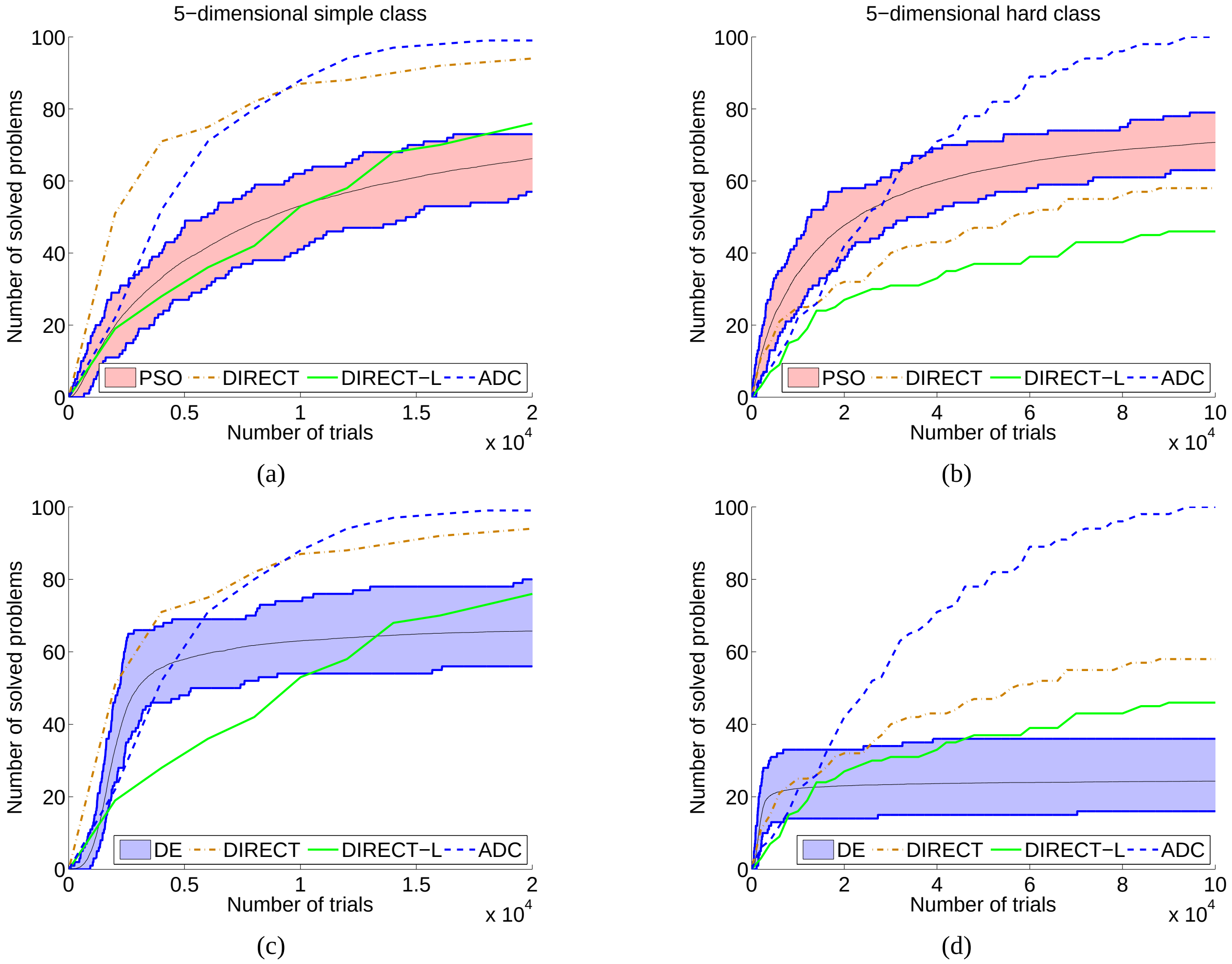

Figure 7. Operational characteristics and operational zones for the 5-dimensional GKLS classes for metaheuristics PSO and DE. **a,** Operational characteristics for deterministic methods and operational zones for PSO are presented for the simple class. **b,** The same as (a) for the hard class. **c,** Operational characteristics for deterministic methods and operational zones for DE are presented for the simple class. **d,** The same as (c) for the hard class.

Table 2. Results of the experiments. For each test class the average number of trials required to solve all 100 problems is presented for each deterministic algorithm. For each metaheuristic method, the average number of trials required to solve each problem on 100 runs has been calculated, and the average of these 100 values is presented. †

N	Class	**Metaheuristic algorithms** (10000 runs for each algorithm and class)		**Deterministic algorithms** (100 runs for each algorithm and class)		
		Differential Evolution	Particle Swarm Optimization	DIRECT	DIRECT-L	Diagonal Algorithm
2	simple	>52910.38(511)	>110102.74(1046)	198.9	292.8	176.3
2	hard	>357467.49(3556)	>247232.35(2282)	1063.8	1267.1	675.7
3	simple	>165125.02(1515)	>170320.10(1489)	1117.7	1785.7	735.8
3	hard	>476251.20(4603)	>285499.04(2501)	>42322.7(4)	4858.9	2006.8
4	simple	>462401.52(4546)	>303436.36(2785)	>47282.9(4)	18983.6	5014.1
4	hard	>773481.03(7676)	>456996.08(4157)	>95708.3(7)	68754	16473
5	simple	>294839.01(2815)	>181805.17(1561)	>16057.5(1)	16758.4	5129.9
5	hard	>751930.00(7473)	>250462.63(2109)	>217215.6(16)	>269064.4(4)	30471.8

†The record “>m(i)” means that the algorithm did not solve a global optimization problem *i* times in 100 runs ×100 problems (i.e., in 10,000 runs for metaheuristics and in 100 runs for deterministic algorithms). In this case, the maximal number of trials set to 10^6 was used to calculate the average number of trials *m*.
